# Comparison of choroidal thickness and choroidal vascular index in normotensive dippers and nondippers

**DOI:** 10.1590/1806-9282.20230950

**Published:** 2024-03-15

**Authors:** Doğukan Cömerter, Taha Baysal, Selami Doğan, Almina Erdem, Tufan Çınar

**Affiliations:** 1 University of Health Sciences, Sultan Abdülhamid Han Training and Research Hospital, Department of Ophthalmology – Istanbul, Turkey.; 2 University of Health Sciences, Sultan Abdülhamid Han Training and Research Hospital, Department of Cardiology – Istanbul, Turkey.

**Keywords:** Optical coherence tomography, Disease, choroidal, Ambulatory blood pressure monitoring, Hypertension

## Abstract

**OBJECTIVE:**

The aim of this study was to evaluate the choroidal thickness and choroidal vascular index in normotensive individuals with dipping and nondipping patterns.

**METHODS:**

Patients who applied to the cardiology clinic for routine checkups and underwent 24-h blood pressure monitoring were included in our study. They were divided into two groups based on their dipper status. The patients in whom systolic blood pressure decreased during the nocturnal time by 10% or more of the daily blood pressure were defined as dippers. On the contrary, patients whose nocturnal systolic blood pressure decreased by less than 10% were defined as nondippers. Choroidal thickness and choroidal vascular index were measured by spectral-domain optical coherence tomography. Central macular thickness, retinal nerve fiber layer, and ganglion cell layer (GCL) analyses were also recorded.

**RESULTS:**

In total, 35 patients with dipper pattern and 34 patients with nondipper pattern were recruited. The mean subfoveal choroidal thickness was 349.72±90 μm in the dipper group and 358.54±132.5 μm in the nondipper group. The groups had no significant difference in choroidal thickness, central macular thickness, retinal nerve fiber layer, and ganglion cell layer analyses. However, the choroidal vascular index was statistically significantly lower in the nondipper group when compared to the dipper group (0.61±0.02 vs. 0.64±0.02; p<0.001). Also, the choroidal vascular index was negatively correlated with subfoveal choroidal thickness in the nondipper group (Spearman; r=-0.419; p=0.033).

**CONCLUSION:**

Our study showed that the choroidal vascular index was significantly lower in nondippers than in dippers. Nondipper individuals may be affected by vascular dysregulation, leading to alterations in the choroidal circulation.

## INTRODUCTION

Increased arterial blood pressure, also known as hypertension (HT), is a major risk factor for coronary heart disease, stroke, and renal failure^1^. Early and consistent detection of subtle microvascular changes in prehypertensive patients may provide prognostic information for cardiovascular risk stratification and disease progression^2^. Cardiovascular parameters, such as BP and heart rate (HR), change with the circadian rhythm throughout the day. Some studies show that both BP and HR in normotensive subjects decrease and remain relatively low throughout the night and then rise precisely in the early morning hours to reach a peak^3^. Patients with BP that does not decrease by 10% during night sleep compared to daytime are defined as "nondippers". Target organ injury, defined as clinical or laboratory finding of early hypertensive damage in any vascular organ, occurs more severely in nondipper hypertensive patients than in dipper patients^4^. Ambulatory blood pressure monitoring (ABPM) is commonly used to detect the dipper or nondipper pattern in BP readings^5^. The 24-h ABPM to identify dipping or nondipping pattern has become increasingly crucial for managing patients with pre-HT or essential HT^6^.

The eye is a critical organ reflecting hypertensive microvascular effects and allows direct observation. Evaluation of visual parameters may provide a predictive and prognostic value in managing systemic complications secondary to HT, diabetes mellitus, and cardiovascular, cerebrovascular, and other systemic vascular diseases^7^. With the advances in imaging technology, SD-OCT provides visualization and measurement of the retinal layers and choroidal thickness (ChT). New OCT image modalities, including enhanced-depth imaging mode OCT (EDI-OCT) and optical coherence tomography-angiography (OCT-A), enable better visualization of the choroid and vascular plexuses in contrast to conventional techniques^8^.

Several reports claim that changes in the microvascular structure of the choroid can be a sign of a systemic disease that affects blood vessels. Thus, the relationship between choroid and cardiovascular diseases (CVD) becomes an important clinical entity^9^. In the current literature, a prior study investigated the ChT in hypertensive patients with nondipper and dipper patterns and found significant differences among these patients^10^. As early identification of vascular changes in normotensive patients with nondipper status may have an impact on the prognosis of such patients, this study aimed to compare ChT and CVI changes in normotensive dippers and nondippers.

## METHODS

### Study participants

This study was designed as a prospective, single-center study. The local ethics committee approved the study protocol and conducted it following the tenets of the Declaration of Helsinki (HNEAH-KAEK-2023/1106-4229). Informed consent was acquired from each subject before enrollment in the study.

The study group comprised 69 patients who applied to the cardiology clinic for the evaluation of blood pressure and were found to be normotensive based on 24-h AMBP monitoring. Patients with coexisting diseases such as diabetes mellitus, coronary artery disease, carotid artery stenosis, heart failure, renal failure, stroke, debilitating illness, hyperlipidemia, and dementia as well as patients with BMI>25 kg/m^2^ were excluded from the study. In addition, patients with any ocular diseases (glaucoma, uveitis, and other retinal and neurodegenerative diseases), history of previous retinal treatment (laser photocoagulation and intravitreal injection), any intraocular surgery other than uneventful phacoemulsification, and media opacities that obscured the choroidal imaging were not included in the study.

### Study protocol and procedure

Hypertension was defined as systolic blood pressure (SBP) >140 mmHg and/or diastolic blood pressure (DBP) >90 mmHg, according to the 2018 European Society of Hypertension/European Society of Cardiology Guidelines for the management of arterial HT^11^. Based on the results of 24-h ABPM, patients who were found to be normotensive were classified into two groups according to their dipper status. Compared to daytime values, those whose night-time SBP decreased ≥10% were defined as dippers, and those whose SBP decreased <10% were defined as nondippers. Ophthalmic examination and SD-OCT imaging were performed by the same physician (D.C.) in a blinded manner. Ophthalmic examination included best-corrected visual acuity (BCVA) with Snellen chart, anterior segment evaluation with slit-lamp biomicroscopy, intraocular pressure (IOP), and funduscopic examination. All study participants had a BCVA of 20/20 and IOP lower than 21 mmHg. OCT images were obtained with the Spectralis OCT with eye-tracking dual-beam technology (Heidelberg Engineering GmbH, Heidelberg, Germany). One eye was randomly selected as the study eye. The fellow eye was studied if the randomly selected eye did not meet the inclusion criteria. CMT, RNFL, and GCL analyses were also recorded.

Choroidal thickness was measured manually using the caliper provided by EDI-OCT as the perpendicular distance between the hyperreflective outer border of the retinal pigment and the epithelial – Bruch's membrane – layer. The ChT was obtained at five different points in a horizontal scan line: the subfoveal, 500 μm and 1500 μm temporal to the fovea, and 500 μm and 1500 μm nasal to the fovea.

Choroidal vascular index was calculated using the ImageJ software (version 1.50a; NIH, Bethesda, MD, USA). The choroidal area (CA) was measured in a total of 3000 μm area (margin of 1500 μm nasal and temporal to the fovea center) horizontally and from the retina pigment epithelium (RPE) to the choroidoscleral border vertically. The edges of the CA were identified manually using the ImageJ ROI Manager. Binarization was executed using the Niblack auto-local threshold method. Dark pixel areas represent the vascular channels (luminal area, LA), and light pixel areas represent the stroma of the choroid (stromal area, SA) in the binarized image. CVI was calculated as the proportion of the LA to the total CA. Manual measurements were performed by the same physician (D.C) who is blinded to the groups. Measurements with a difference of more than 10% were excluded from the study.

### Statistical analysis

Statistical analysis was analyzed by the SPSS program for Windows version 22. The Kolmogorov-Smirnov test was performed to determine whether continuous variables were distributed normally. The independent samples t-test and Mann-Whitney U-test were used to compare the quantitative data. For correlation analysis, the Spearman correlation analysis test was used. All values are given as means±standard deviations, and significance was considered p≤0.05.

## RESULTS

This study included 35 patients (17 males and 18 females) with dipper patterns and 34 (14 males and 20 females) with nondipper patterns. The demographic and clinical characteristics of the subjects were similar at the baseline. There was no significant difference in the mean age, gender, daytime SBP, daytime DBP, night-time SBP, night-time DBP, and total BP values among the groups. As expected, the night-time SBP was higher in the nondipper group (115.85±11.1 mmHg vs. 112.64±9.9 mmHg). However, there was no statistically significant difference in BP parameters between the groups. BP parameters are shown in Table 1.

**Table 1:** Comparison of total, daytime, and night-time blood pressure in dipper and nondipper patients.

BP parameters (mmHg)	Dipper (n=35)		Nondipper (n=34)		p
	min–max	Mean±SD	min–max	Mean±SD	
Systolic BP, total	100–141	120±10.6	92–137	120.08±10.4	0.979
Diastolic BP, total	59–88	75.64±8.6	59–86	72.85±7.8	0.229
Systolic BP, daytime	100–136	121.08±10.4	96–140	120.92±10.1	0.957
Diastolic BP, daytime	60–90	76.8±9.3	61–94	75.35±8.7	0.566
Systolic BP, night-time	98–129	112.64±9.9	97–138	115.85±11.1	0.283
Diastolic BP, night-time	55–85	68.92±8.8	50–85	67.85±9.1	0.67

The mean subfoveal ChT was 349.72±90 μm in the dipper group and 358.54±132.5 μm in the nondipper group. There was no significant difference between the groups in ChT, CMT, RNFL, and GCL analyses. However, subfoveal ChT was positively correlated with daytime DBP (Spearman; r=0.464; p=0.017). OCT measurements are summarized in Table 2.

**Table 2:** Comparison of choroidal thickness, ganglion cell layer thickness, central macular thickness, and retinal nerve fiber layer thickness in dipper and nondipper patients.

Parameters	Dipper group (n=35)		Nondipper group (n=34)		p
	min–max	Mean±SD	min–max	Mean±SD	
Subfoveal ChT (μm)	203–566	349.72±90	131–610	358.54±132.5	0.992
ChT, nasal (500 μm)	140–561	345.6±104.8	96–581	362.46±133.8	0.62
ChT, nasal (1500 μm)	127–552	282.48±106.2	106–509	314±126.7	0.318
ChT, temporal (500 μm)	189–561	345.12±86.5	140–638	355.35±131.3	0.743
ChT, temporal (1500 μm)	200–552	315.08±89.9	107–561	328.73±111.4	0.559
GCL thickness (μm)	7–28	14.84±4.9	9–28	14.77±5.1	0.82
CMT (μm)	201–272	232.76±19.2	189–304	224.69±27	0.124
RNFL thickness (μm)	82–130	103.4±11.5	59–118	96.31±13.1	0.113

The CVI was statistically significantly lower in the nondipper group when compared to the dipper group. (0.61±0.02 vs. 0.64±0.02; p=0.0001). Additionally, the CVI was significantly negatively correlated with the subfoveal ChT in the nondipper group (Spearman; r=-0.419; p=0.033<0.05). A positive correlation was also found between CVI and night-time DBP when all participants were considered together (Spearman; r=0.301; p=0.032<0.05). Table 3 shows the relationship between subfoveal ChT, CVI, and BP values.

**Table 3:** Correlation between the choroidal vascular index, blood pressure, and subfoveal choroidal thickness.

CVI		Subfoveal ChT	Systolic BP, total	Diastolic BP, total	Systolic BP, daytime	Diastolic BP, daytime	Systolic BP, night-time	Diastolic BP, night-time
All participants (n=69)	r	-0.355	0.151	0.260	0.138	0.079	0.140	0.301
	p	0.011*	0.291	0.066	0.335	0.580	0.326	0.032*
Dipper group (n=35)	r	-0.304	0.295	0.199	0.242	0.061	0.368	0.430
	p	0.139	0.153	0.341	0.243	0.774	0.071	0.032*
Nondipper group (n=34)	r	-0.419	0.201	0.296	0.181	0.096	0.231	0.309
	p	0.033*	0.324	0.142	0.377	0.640	0.257	0.124

* indicates statistical significance ones.

## DISCUSSION

Choroid circulation has one of the highest rates of blood flow in the human body^12^. Choroid supplies oxygen and nutrients to the retinal layers between RPE and up to the inner nuclear layer^13^. Therefore, healthy choroidal vasculature is essential for normal functioning of the retina. Choroidal arteries have a unique structure in the choriocapillaris. Due to this morphology, high BP is transmitted directly to the choriocapillaris, and choroidal vessels are capable of blood flow autoregulation in response to changes in BP.

This study demonstrated the choroidal stromal and vascular changes in normotensive individuals with anomalous circadian BP patterns. Several previous studies have shown a better understanding of the vascularity of the choroid in both healthy and diseased eyes using SD-OCT^14-16^. However, previous studies mainly focused on ChT in cardiovascular diseases or eye disorders^17,18^. Tas et al.^10^ reported that ChT in subfoveal and temporal locations were lower in the nondipper group, and they also found a negative correlation between night-time SBP and ChT. They focused on hypertensive patients with/without dipping patterns. In our study, we want to evaluate ChT and CVI in normotensive individuals with these patterns. We found that CVI values were statistically significantly lower in nondippers than in dippers. Moreover, a negative correlation was detected between CVI and subfoveal ChT in the nondipper group. We may explain these results with vasoconstriction and chronic Ischemia of vascular plexuses when anomalous BP patterns exist for a long time. Vasoconstriction in choroidal vessels, Ischemia, and RPE changes may play an essential role in the pathophysiology and progression of many choroidal and retinal diseases. Also, these findings suggest that CVI may be a more stable and reliable index for vascular status in CVD when compared to ChT.

Previous studies have described the thickening and thinning of the subfoveal choroid in the presence of CVD risk factors such as diabetes and hypertension^19-21^. The complex physiology of the choroidal vasculature, the impact of disease characteristics and medications, and even the retinal vasculature's status may complicate the ChT assessment in systemic diseases^22^. While Ahn et al.^23^ found a significant correlation between the ChT and BP, other studies did not^24^. In our present study, we found no significant difference in BP parameters (daytime or night-time SBP and DBP). Also, no correlation was found between the ChT and BP values in all subjects; however, only subfoveal ChT was positively correlated with daytime DBP in the nondipper group. Gök et al.^20^ reported that subfoveal ChT did not differ significantly between the dipper and nondipper hypertensive groups. Similarly, we found no significant difference in ChT measurements in normotensive individuals with dipping or nondipping patterns.

Lee et al., reported that the RNFL/GCL thickness ratios of the patients with chronic HT did not differ from the normal controls. In the other study, the RNFL thickness of hypertensive patients was thinner than healthy controls, which was most prominent in the superior and inferior quadrants^25^. In our present study, no significant differences in CMT, RNFL, and GCL were found between the groups. These inconsistent results may be due to undeveloped HT or the need for time to cause end-target organ damage.

Our study has some limitations, which must be considered when assessing our results:

1. Our sample size was small.
2. Since all of our study participants were Caucasians, our conclusion should be considered valid for this ethnicity only and cannot be generally applied to other ethnicities or genetic backgrounds.
3. The manual transmission of all identities of the Bruch's membrane and the inner scleral border, as Heidelberg SD-OCT equipment, did not automatically segment the choroid.

## CONCLUSION

This is the first clinical study evaluating ChT and CVI in normotensive subjects with dipping and nondipping patterns. Our study showed that CVI was significantly lower in nondippers than in dippers. We found no difference in ChT between the groups. We discover that these anomalous BP patterns could affect choroidal changes in patients with CVD and should be considered when ChT and CVI are evaluated for chorioretinal diseases or other clinical studies. Larger, prospective studies will be needed to confirm our preliminary results, in particular, to clarify the role of nocturnal BP and nondipping patterns in normotensive or hypertensive subjects.
